# Understanding the situation of vegans

**DOI:** 10.1007/s40519-021-01127-2

**Published:** 2021-02-25

**Authors:** Christian Koeder

**Affiliations:** Department of Nutritional Sciences, University of Applied Sciences Münster, Corrensstr. 25, 48149 Münster, Germany

Dear Editor-in-Chief,

Several recent articles in Eating and Weight Disorders [1, 2] have reported the levels of “orthorexic tendencies” in vegans, as assessed by the Düsseldorf Orthorexia Scale (DOS). Even though the definition of orthorexia nervosa (ON) is controversial, ON is generally understood to be an obsession with healthy eating [1]. Consequently, a high DOS score is interpreted to be undesirable.

Vegans may score higher on the DOS (and other ON scores) [1–3]. Consequently, some researchers have concluded that: “Vast research exists regarding ON’s relationship with vegan and vegetarian patterns, showing that individuals with these patterns have [a] greater tendency to develop ON” [1]. But this interpretation may be flawed. If so, it may pathologize and stigmatize vegans [3]. Therefore, I would like to ask researchers in this field to make an effort to understand and to know vegans better. This may help reduce the risk of drawing erroneous conclusions.

Some of the DOS’s items seem to be based on the assumption that a typical western, non-vegetarian diet is “normal”. But this may conflate two different meanings of the word “normal”: typical and healthy. This may lead to the erroneous conclusion that “untypical” amounts to “unhealthy”. This in turn would make the DOS biased.

A bias against vegans seems most obvious in item 2 of the DOS: “I have certain nutrition rules that I adhere to”. This item is based on the assumption that dietary rules indicate orthorexic tendencies. All vegans have the dietary rule of being dietary vegans. Thus vegans will score higher, seemingly indicating orthorexic tendencies. This is circular reasoning. In addition, vegans may also have other “nutrition rules” such as taking vitamin B12 supplements.

Item 1: “Eating healthy food is more important to me than indulgence/enjoying the food”. Vegans may on average be more health conscious and may then see health more as a means to an end (pleasure of all kinds) and may therefore score higher.

Item 3: “I can only enjoy eating foods considered healthy”. This may indicate a belief that it is extremely important to eat only healthy foods. Many individuals become vegans because of a preexisting illness (diabetes, skin conditions, a heart attack, etc.). They may have been advised to follow their diet strictly. This advice may be evidence-based or it may not be. But being misinformed or following advice strictly does not necessarily indicate an obsession.

Item 4: “I try to avoid getting invited over to friends for dinner if […] they do not pay attention to healthy nutrition”. Vegans may conflate “healthy” with “vegan”. It should also be noted that being vegan may indeed lead to conflict with friends or family. Avoiding an invitation may indicate the intention to avoid unpleasant discussions (about nutrition or animal rights). It may also be that the inviting parties, even if they are friends or family, are to some extent perceived as political “enemies” (the 1980s slogan “meat is murder” is not an empty phrase for many vegans but rather quite accurately reflects their view on eating animal products).

Item 5: “I like that I pay more attention to healthy nutrition than other people”. If vegans are more health conscious than the general population, vegans will likely score higher.

Item 6: “If I eat something I consider unhealthy, I feel really bad”. (German version: “If I have eaten something unhealthy, then I feel guilty”; literally: “[…] I blame myself”) The German version indicates a feeling of guilt. In contrast, the English version may also be understood as feeling physically bad. Again, some vegans may conflate health and ethics. Near-vegans who may occasionally not be vegan may feel guilty because of the animal suffering involved. Some individuals may feel physically “bad” after eating particular foods (e.g., an extremely sugary cupcake), and vegans on average may be more aware of such effects.

Item 7: “I have the feeling of being excluded by my friends and colleagues due to my strict nutrition rules”. Vegans are often excluded. It may be that friends/colleagues do not know how to cater for a vegan, or they may not want to, or they may anticipate unwelcome discussions. Even if everyone at the table aims for the highest level of courtesy and even if there is no mention of animal rights or veganism, the vegan at the non-vegan table remains what has been called the “absent referent”, reminding the others where animal products come from.

Item 8: “My thoughts constantly revolve around healthy nutrition and I organize my day around it”. Vegans may want to plan their diets appropriately and may be more likely to prepare foods in advance because there may be no satisfying vegan options available at work or school.

Item 9: “I find it difficult to go against my personal dietary rules”. Many vegans will affirm this because they would indeed find it difficult or even morally abhorrent if they did.

Item 10: “I feel upset [downcast] after eating unhealthy foods”. This is similar to item 6. However, the German version emphasizes guilt in item 6 and sadness in item 10.

These aspects may explain some of the differences between vegans’ and typical eaters’ DOS scores. In addition, it may be useful for researchers to be aware that vegans are a diverse group, that self-defined vegans are a larger group than actual dietary vegans, that a vegan diet does not equal lower dietary variety or quality, that confounders (especially sex) should be adjusted for, and that stigmatizing minority groups, including vegans, may cause serious harm.

While it is useful to distinguish between “health vegans” and “ethical vegans”, this may not be enough: two-sided questions should be asked instead of trying to confirm prejudices. Null findings may be the result of confounding. When minority groups are assessed, it may be useful to involve them in creating appropriate assessment tools. More research is needed and a better understanding of vegans may be helpful.
